# Targeting Protein Disorder for the Remediation of Antimicrobial Resistance

**DOI:** 10.1021/acsomega.4c08427

**Published:** 2024-12-10

**Authors:** Jack O’ Callaghan, Michael P Ryan, Sarah Hudson, Damien Thompson

**Affiliations:** † Department of Physics, Bernal Institute, University of Limerick, Limerick V94 T9PX, Ireland; ‡ Bernal Institute, University of Limerick, Limerick V94 T9PX, Ireland; § Science Foundation Ireland Research Centre for Pharmaceuticals (SSPC), University of Limerick, Limerick V94 T9PX, Ireland; ∥ Department of Applied Sciences, TUS Midwest, Limerick V94 EC5T, Ireland; ⊥ Department of Chemical Sciences, Bernal Institute, University of Limerick, Limerick V94 T9PX, Ireland

## Abstract

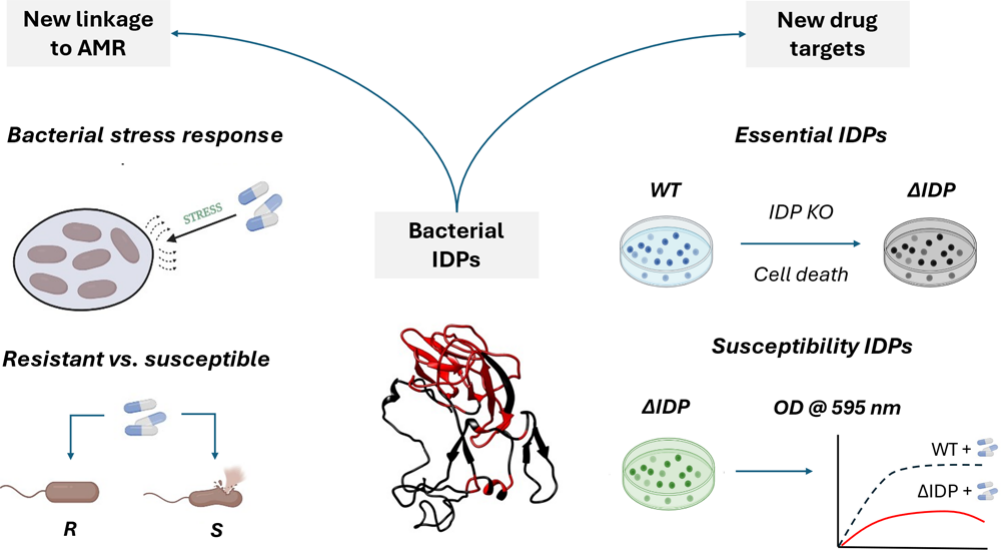

The remediation of antimicrobial resistance (AMR) is a fundamental challenge for global healthcare. Intrinsically disordered proteins (IDPs) are recognized drug targets for neurodegeneration and cancer but have not been considered to date for AMR. Here, a novel link between structural disorder and AMR is identified by mapping predicted disorder profiles onto existing transcriptomic data for resistant and susceptible *E. coli* isolates. The AMR-relevant IDPs fall into two distinct classes, those involved in the bacterial stress response and those differentially expressed between resistant and susceptible strains following antibiotic exposure. A residue-wise conservation analysis of relevant bacterial IDPs identified mutations within intrinsically disordered regions that correlate with pronounced changes in antimicrobial susceptibility, providing valuable insight into the functional importance of bacterial intrinsic disorder in the ESKAPEE pathogens. The identification of susceptibility-inducing IDPs in *E. coli* highlights the potential of disorder-based antimicrobial drug discovery for the remediation of drug-resistant bacterial infections.

## Introduction

Bacteria exploit diverse mechanisms to subvert the action of antimicrobial agents, fuelling the emergence of antibiotic-resistant strains that are challenging to treat.^1^ In particular, the ESKAPEE pathogens^2^ are highly prioritized by the World Health Organization (WHO) for the development of new treatments. These pathogens encompass seven highly virulent and resistant bacteria: *Enterococcus faecium*, *Staphylococcus aureus*, *Klebsiella pneumoniae*, *Acinetobacter baumannii*, *Pseudomonas aeruginosa*, *Enterobacter* spp., and *Escherichia coli*. Novel strategies are being developed to tackle antimicrobial resistance (AMR) including innovations in antimicrobial drug target discovery,^3^ the deep learning-led design of novel antibiotics,^4^ and machine learning (ML)-guided drug repurposing.^5^

In an effort to expand the search space for target identification, we explore here the possible role of bacterial proteomic disorder in remediating AMR. Intrinsically disordered proteins (IDPs) are biological macromolecules that lack a well-defined secondary or tertiary structure under normal physiological conditions. In contrast to ordered, well-structured proteins, IDPs are enriched with charged amino acids. This can give rise to intramolecular electrostatic repulsion effects that preclude the formation of secondary structures.^6^ IDPs reflect a class of drug targets not previously considered in the context of AMR. However, the evolution of AMR is innately linked to the bacterial stress response,^7^ and disordered stress effectors have previously been reported in nonprokaryotic systems.^8^ In the present work, the relationship between bacterial intrinsic disorder and antimicrobial resistance was investigated. First, disorder profiles for representative bacterial strains from the ESKAPEE pathogens were constructed. As a test case to investigate the relationship between IDPs and AMR, the transcriptional response of resistant and susceptible bacteria to antibiotic-induced stress in *E. coli* was then explored. A structural analysis of relevant IDPs identified mutations in intrinsically disordered regions (IDRs) that directly correlate with substantive changes in antimicrobial susceptibility.

**Figure 1:**
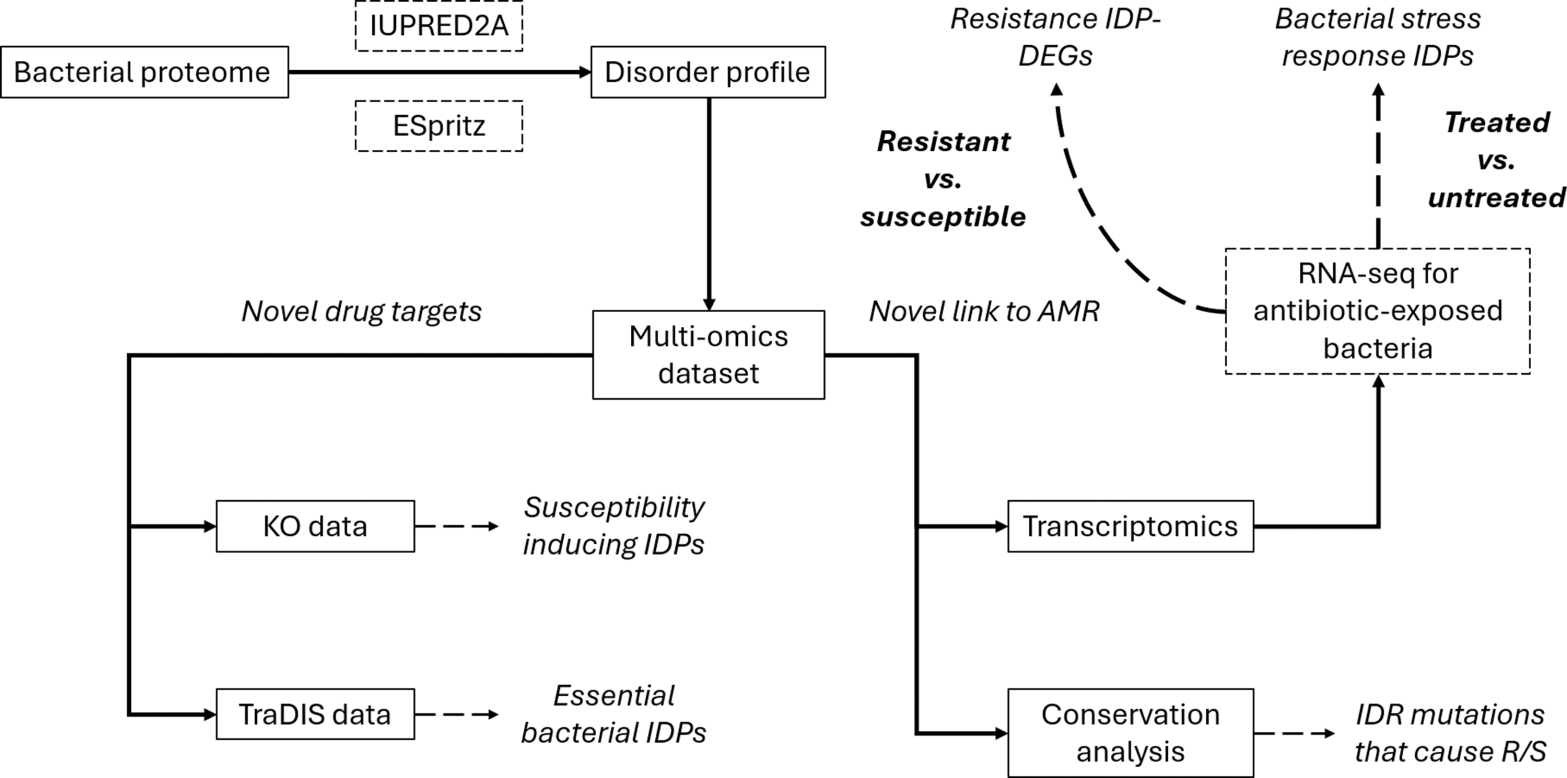
Summary of the novel methodology developed to test the hypothesis that bacterial IDPs are implicated in AMR evolution. Outputs are marked by dashed arrows.

Overlaying IUPRED2A-derived^9^ or ESpritz-derived^10^ disorder profiles with single-gene knockout (KO) data reveals IDPs that cause antimicrobial susceptibility, and transposon-directed insertion site sequencing (TraDIS) data can be mined to identify essential bacterial IDPs. Combining residue-wise conservation scores with relevant disorder profiles can discern mutations in bacterial IDRs that mediate AMR or are otherwise associated with an increase in resistance (R) or susceptibility (S). RNA-sequencing (RNA-seq) data can be used to quantify changes in gene expression following antibiotic exposure. Bacterial IDP-encoding genes differentially expressed in response to stress or differentially expressed between resistant and susceptible isolates support a novel relationship between intrinsic disorder and AMR (Figure 1).

## Methods

### Data Collection

Bacterial proteomic data sets were sourced from UniProt.^11^ Next-generation sequencing (NGS) data was sourced from the European Nucleotide Archive (ENA).^12^ When constructing disorder profiles for ESKAPEE pathogens, proteomes were selected from UniProt if they (1) belonged to the ESKAPEE pathogens and (2) were classed as a reference proteome. Additional proteomes were selected if they (1) belonged to the ESKAPEE pathogens, (2) were not classed as redundant, or (3) exhibited a high degree of data completeness. This degree of completeness was determined using two integrated UniProt tools: Benchmarking Universal Single-Copy Ortholog (BUSCO) and Complete Proteome Detector (CPD). The application of these criteria yielded 58 proteomes.

### Profiling Regions of Intrinsic Disorder in Bacterial Proteins

Custom AWK, R, and Python scripts were written to automate the construction of a database quantifying the disorder in each bacterial proteome. As an input, this analysis pipeline takes a multi-FASTA (.fa) file relating to the bacterial proteome of interest (obtained from UniProt). An AWK script is used to parse the input multi-FASTA file into individual FASTA files for each protein. Each file is named after the gene that identifies it, as determined from the syntax and organization of the FASTA file. A Python script then applies the predictive software IUPRED2A^9^ to all FASTA files, generating a disorder profile for each protein. This profile is outputted as a text (.txt) file and includes (1) the position of a given residue, (2) the one letter amino acid symbol, (3) a Z-score used to determine if a given residue is disordered, and (4) a Z-score used to determine if a given residue is involved in binding. Disordered binding residues define a region of a protein that can (1) form favorable interactions with the binding surface of another protein and (2) are embedded in a generally disordered sequence environment. An R-script is then used to store each disorder profile as a data frame. This list of data frames is analyzed, and various parameters are calculated (Table S1). These outputs are then consolidated into a single data frame, ordered alphabetically (according to the gene name), and then written to a CSV (.csv) file. The outputted CSV file is a database with information on the disorder of each protein in the proteome, hereafter referred to as “whole-proteome profiling”.

### Time Series Analysis for Bacterial RNA-Seq Data

RNA-sequencing data for antibiotic-exposed bacteria was obtained from the study of Bhattacharyya et al.^13^ in order to identify IDPs involved in the bacterial stress response and the evolution of AMR in *E. coli*. For each antibiotic, gene expression data was recorded for two clinical isolates that were resistant (R) and for two clinical isolates that were susceptible (S). RNA-seq libraries were prepared at four different time points of 0, 10, 30, and 60 min postantibiotic exposure for each antibiotic–strain combination. Relevant information for each experimental run is detailed in Table 1.

**Table 1:** Experimental Conditions Used to Treat Two Resistant (R) and Two Susceptible (S) Clinical *E. coli* Isolates^a^

antibiotic	minimum inhibitory concentration (MIC, mg/L)			
	*Strain RB051 (R)*	*Strain RB057 (R)*	*Strain RB001 (S)*	*Strain RB075 (S)*
ciprofloxacin (1 mg/L)	64	64	0.03	0.03
gentamicin (4 mg/L)	256	256	1	0.5

a Conditions taken from Bhattacharyya et al.^13^

In the context of transcriptome profiling data for antibiotic-exposed bacteria, differentially expressed genes (DEGs) reflect genes with a statistically significant difference in the abundance of associated gene transcripts postantibiotic exposure. Statistically significant DEGs were calculated using the R-package DeSeq2^14^ and visualized using the Bioconductor package EnhancedVolcano.^15^ Constraints of a minimum log-fold change of |log_2_FC| > 1 and a minimum statistical significance of *p*_adj_ < 0.10 were imposed to identify relevant DEGs. Two models were applied (via DeSeq2) in order to assess the differential expression of IDP-encoding genes between resistant and susceptible strains. When evaluating expression changes across more than two levels (i.e., for differential gene expression analysis across multiple time points), a reduced model of the likelihood ratio test (LRT) was used. DEGs were also calculated between susceptible and resistant bacteria at specific time points. In the case of pairwise comparisons, the default Wald test was used.

### Predicting Evolutionary Conservation in Bacterial Proteins

Examining the evolutionary dynamics of amino acid substitutions between homologous sequences is an established method of predicting functional regions in proteins.^16^ Here, the sequence conservation of selected IDPs was calculated utilizing a combination of the NCBI basic local alignment search tool (BLAST^17^), constraint-based local alignment tool for multiple protein sequences (COBALT^18^), multiple sequence alignment software (MUSCLE^19^), and ConSurf.^20^ These IDPs were selected based on a literature review of IDP-DEGs calculated from the response of *E. coli* K12 to 2 mg/L of ciprofloxacin (Figure S1). When using BLAST, search sets were derived from standard databases, primarily the UniProt Knowledge Base (UniProtKB). The algorithm was run with 100 maximum target sequences, using an “expect cutoff” threshold of α = 0.05, and scored using the BLOSUM62 matrix with conditional compositional score matrix adjustment. When the position-specific iterated variant of BLAST was used (PSI-BLAST, from which the COBALT results are derived), 500 target sequences were used. Two iterations of PSI-BLAST were also carried out at a threshold value of α = 0.005 to support the computation of conservation scores. The default settings were used for both ConSurf and MUSCLE. For ConSurf, in cases where there were initially insufficient homologous sequences to establish statistically significant conservation scores, the number of target sequences was increased from 150 to 300.

## Results and Discussion

### Disorder Profiles of Relevant ESKAPEE Pathogens

**Figure 2:**
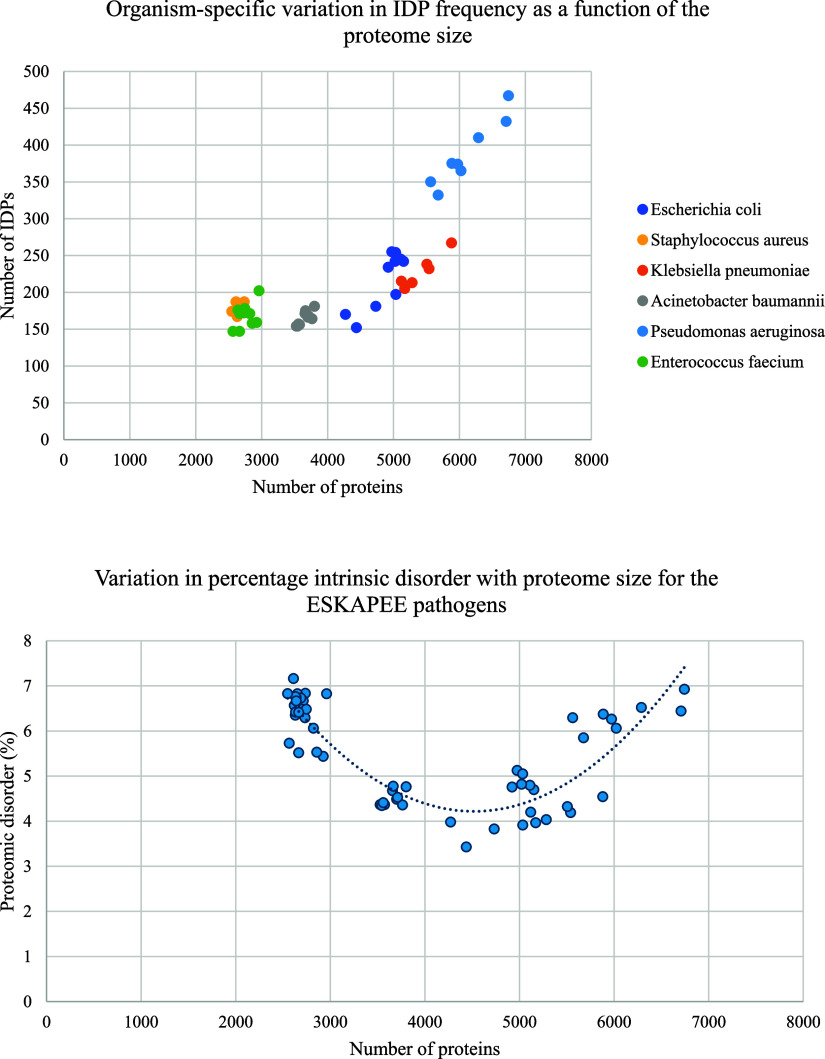
Top panel: graphical summary of whole-proteome profiling analysis across 58 unique strains of the ESKAPEE pathogens. Bottom panel: percentage of disordered proteins vs proteome size across 58 unique strains of the ESKAPEE pathogens.

In order to generate a foundational data set with which to assess bacterial intrinsic disorder in a multiomics context, whole-proteome profiling was performed with respect to intrinsic disorder for 58 strains across 6 representative microorganisms of the clinically relevant ESKAPEE pathogens. These strains were selected as they constituted a complete set of nonredundant proteomes spanning the six pathogens under consideration. This analysis entailed a context-dependent prediction of disordered residues in 232,422 proteins using the software IUPRED2A.^9^ Proteins were classified as IDPs if they contained “long” IDRs, defined as a continuous disordered region greater than 30 amino acids in length.^21^ A summary of this profiling is presented in Figure 2 and Table 2. This analysis revealed general trends in the abundance of IDPs across different bacterial species. Specifically, the number of IDPs increased with an increase in proteome size (beyond a threshold of ∼4500 proteins), and a parabolic relationship was observed between the percentage of IDPs in the proteome and the proteome size (with a similar inflection point at ∼4500 proteins, Figure 2). Proteomic disorder among the ESKAPEE pathogens was found to be between 3 and 7%.

**Table 2:** Scope of Whole-Proteome IDP Profiling Analysis for the ESKAPEE Pathogens Using IUPRED2A

organism	strain		UniProt accession	number of proteins	number of IDPs	percentage of IDPs (%)
*Escherichia coli*	K12	MG1655	UP000000625	4438	152	3.42
Gram negative (−ve)		W3110	UP000000318	4273	170	3.98
	O157:H7	DEC5D	UP000501131	4733	181	3.82
		DEC5E	UP000501234	5153	242	4.70
		EC869	UP000004641	5111	245	4.79
		EHEC	UP000000558	4976	255	5.12
		MB9–1	UP000509786	5034	254	5.05
		TT12B	UP000372690	4922	234	4.75
		TW18224	UP000562010	5036	197	3.91
		USDA5905	UP000509415	5024	242	4.82
*Klebsiella pneumoniae*	ATCC 700721		UP000304895	5541	232	4.19
Gram negative (−ve)	Ecl8		UP000009316	5172	205	3.96
	HS11286		UP000007841	5284	213	4.03
	MRK9		UP000465088	5120	215	4.20
	R1786		UP000269786	5881	267	4.54
	WBB417		UP000218240	5507	238	4.32
*Acinetobacter baumannii*	AB042		UP000186130	3659	171	4.67
Gram negative(−ve)	AB4568		UP000229219	3702	166	4.48
	ATCC 17978		UP000509446	3666	175	4.77
	ATCC 19606		UP000005740	3765	164	4.36
	DR1		UP000216874	3712	168	4.53
	PHEA-2		UP000179937	3802	181	4.76
	TG29392		UP000272206	3575	156	4.36
	WKA02		UP000192809	3528	154	4.37
	XH647		UP000179937	3802	181	4.76
	XH731		UP000249421	3545	154	4.34
	XH859		UP000070545	3561	157	4.41
*Staphylococcus aureus*	C45		UP000505270	2737	187	6.83
Gram positive (+ve)	CM34		UP000249689	2631	167	6.35
	CN1		UP000016220	2715	181	6.67
	EOE190		UP000250288	2612	187	7.16
	ICE	98	UP000518800	2653	181	6.82
	ISU926		UP000196798	2700	175	6.48
	M013		UP000005441	2627	173	6.59
	MOS462		UP000505220	2690	181	6.73
	MRSA252		UP000000596	2640	178	6.74
	MW2		UP000000418	2633	178	6.76
	N315		UP000000751	2549	174	6.83
	NCTC	8325	UP000005003	2620	172	6.56
	NRS107		UP000035542	2634	169	6.42
*Pseudomonas aeruginosa*	ATCC 15692		UP000002438	5564	350	6.29
Gram negative (−ve)	BL04		UP000017518	6709	432	6.44
	D9	3362	UP000286578	6745	467	6.92
	H47921		UP000063433	6024	365	6.06
	KCJ3K67		UP000480944	6290	410	6.52
	M18		UP000009043	5678	332	5.85
	PA7		UP000001582	5974	374	6.26
	UCBPP	PA14	UP000000653	5886	375	6.37
*Enterococcus faecium*	825		UP000218403	2822	171	6.06
Gram positive (+ve)	ATCC BAA-472		UP000005269	2666	147	5.51
	E3984		UP000438319	2641	176	6.66
	F782		UP000313746	2734	172	6.29
	HM1073		UP000013828	2926	159	5.43
	P3C	A35	UP000271138	2747	178	6.48
	SD3B-2		UP000014603	2858	158	5.53
	UAA1280		UP000013667	2961	202	6.82
	UC8668		UP000027396	2667	171	6.41
	V2937		UP000325664	2567	147	5.73

### Establishing a Novel Link between Bacterial IDPs and AMR

Genes differentially expressed in response to stress can become resistance factors by predisposing physiological changes in cells that promote survival.^7^ This was our motivation for investigating the role played by bacterial IDPs in the microbial stress response. Utilizing RNA-seq data obtained from Bhattacharyya et al.,^13^ the expression of ordered and disordered proteins for clinical isolates of *E. coli* in response to ciprofloxacin (CFX) and gentamicin (GEN) was compared.

In response to 1 mg/L CFX, 22.8% of all proteins (1075 out of 4708) were classed as differentially expressed between antibiotic-exposed and control samples in CFX-susceptible strains (Figure S2). 25.8% of all *E. coli* IDPs (39 out of 151) were among these DEGs. In order to explore the role played by IDP-DEGs in the bacterial stress response, the top 60 differentially expressed genes (ranked according to *p*_adj_) were considered. These DEGs represent the strongest transcriptional signals. Eleven of these DEGs encoded for ordered proteins (*pyrC*, *dinF*, *dsbB*, *queG*, *ubiJ*, *yhdV*, *brnQ*, *rimI*, *soxS*, *ubiE*, and *tomB*), 3 encoded for IDPs (maximum IDR > 30 AAs, *sspB*, *yjgL*, and *rmuC*), and the remaining DEGs encoded for proteins with maximum IDRs < 30 AAs and so could not be classified as IDPs. Bacterial IDPs account for less than 5% of the *E. coli* proteome (see Table 2), yet this analysis revealed that some of the strongest transcriptional signals in response to CFX-induced stress can derive from IDP-encoding genes. It also demonstrates that ordered proteins do not predominate the Top 60 DEGs in response to 1 mg/L of CFX. Separately, transcriptome profiling data relating to the exposure of *E. coli* K12 MG1655 to a higher concentration of CFX (2 mg/L)^22^ were reanalyzed with respect to intrinsic disorder (Figure S1), showing again that genes encoding for nonordered proteins are among the strongest transcriptional signals in response to stress at higher concentrations of CFX (Figure S3).

Similarly for gentamicin (GEN) susceptible strains, in response to 4 mg/L GEN, 20.0% of all proteins (940 out of 4708) were classified as DEGs and 19.2% (29 out of 151) of all *E. coli* IDPs were among these DEGs (Figure S4). Considering the Top 60 DEGs with respect to intrinsic disorder, 6 proteins could be classified as IDPs (*hslR*, *proQ*, *cpxP*, *dnaK*, *ftsH*, and *mutL*), 13 were ordered (*soxS*, *dacA*, *tusB*, *hslV*, *azuC*, *nadK*, *hicB*, *eamA*, *asnC*, *pspG*, *pspC*, *pspB*, and *rcnB*), and the remainder had IDRs < 30 AAs. The differential expression of unstructured proteins in response to stress (via two antibiotics with distinct mechanisms of action) underlies the importance of intrinsic disorder for the bacterial stress response, as it relates to antibiotics. This draws a parallel with the stress biology of both plants^23^ and animals^24^ which employ disordered stress effectors to mitigate against cellular damage.

It has also been shown that changes in gene expression can accompany resistance, acting to offset the biological cost incurred by the acquisition of AMR.^25^ Although the expression signature of an IDP-encoding gene, on its own, is not sufficient to implicate it in antimicrobial resistance, the transcriptome-wide abundance (or absence) of differentially expressed IDPs could shed light on their involvement in AMR evolution. Similar to the identification of stress response DEGs, transcriptome profiling data from Bhattacharyya et al.^13^ were used to compare the expression of IDPs between resistant and susceptible *E. coli* isolates in response to ciprofloxacin (CFX) and gentamicin (GEN). A reduced likelihood ratio test (LRT) model was used to quantify *E. coli* DEGs in response to 1 mg/L of ciprofloxacin and 4 mg/L of gentamicin.

**Figure 3:**
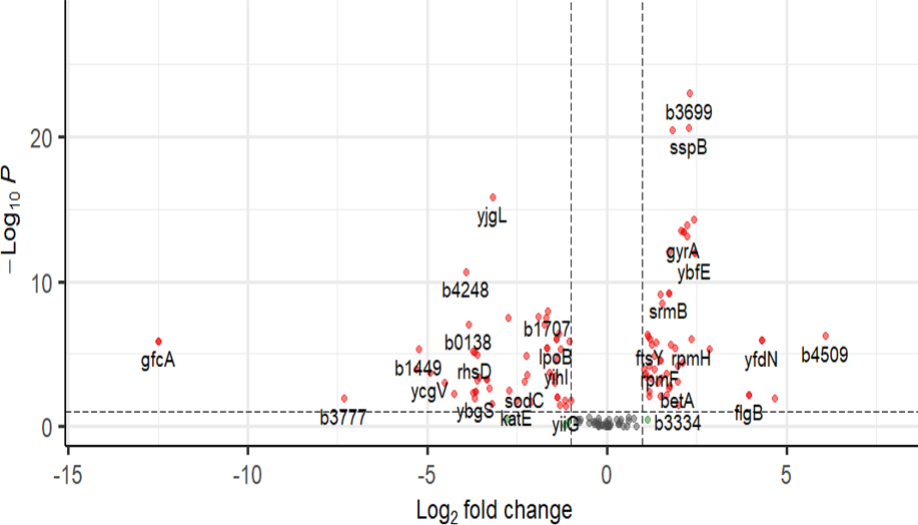
Differential gene expression data for IDP-encoding genes in *E. coli* in response to 1 mg/L of ciprofloxacin.

**Figure 4:**
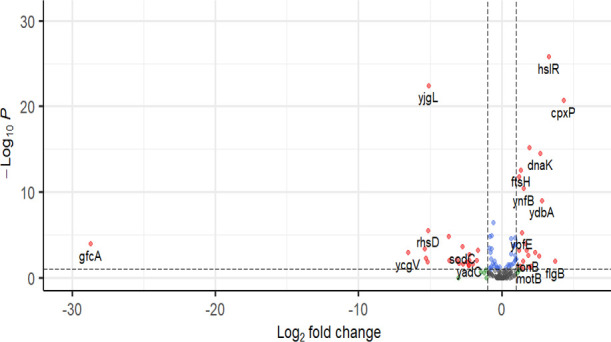
Differential gene expression data for IDP-encoding genes in *E. coli* in response to 4 mg/L of gentamicin.

Leveraging the disorder profile of *E. coli* K12 MG1655 (see Table 2), almost 33% (50 out of 151) of all *E. coli* IDPs were found to be differentially expressed between resistant and susceptible strains following CFX exposure (Figure 3). Repeating this analysis for the exposure of *E. coli* to GEN, 28% (42 out of 151) of all IDP-encoding genes were differentially expressed (Figure 4). The balance between upregulated (UR) and downregulated (DR) IDP-DEGs was approximately equal in response to both CFX (56% DR, 44% UR) and GEN (50% DR, 50% UR). Examining IDPs previously reported to induce antimicrobial susceptibility on knockout (Table 4), four (*rpmF*, *rpsF*, *yih*, and *minC*) were differentially expressed in response to CFX, and five (*ynhG*, *rpsF*, *proW*, *tonB*, and *dnaK*) were differentially expressed in response to GEN. With the exception of *proW*, there was no correlation observed between the differential expression of an IDP-encoding gene and its knockout-induced sensitivity to the antibiotic it was exposed. This indicated that IDP expression profiles were poor biomarkers of both CFX and GEN susceptibility in *E. coli*.

The significance of the differential expression of IDPs between resistant and susceptible *E. coli* isolates is revealed when the relative abundance of IDPs across different bacterial proteomes is considered. As supported by Table 2, bacterial proteomes are typically less disordered than their eukaryotic counterparts.^21^ Despite this lower incidence, a significant number of IDP-encoding genes are differentially expressed between resistant and susceptible strains. To better visualize these IDP-DEGs, functional interaction networks were constructed using the STRING database.^26^ The “multiple proteins” search function was used, and the “list of names” field was populated with the gene names of the IDP-DEGs for the “organism” *Escherichia coli*. These DEGs were found to span putative biological functions and pathways linked to both AMR evolution and bacterial fitness, including ribosomal function^27^ and cell wall biogenesis.^28^ These interaction networks also revealed an abundance of IDPs involved in the functioning of the 50S ribosomal subunit—these include rpmF, rpmH, and rplO (Figure S5).

Using the Wald test, pairwise comparisons were calculated between resistant and susceptible strains at four different time points: 0, 10, 30, and 60 min postantibiotic exposure (Figures S6–S11). In response to both CFX and GEN, a substantive fraction of IDP-encoding genes were differentially expressed during the first 10 min of antibiotic exposure (compared to ordered proteins). Although this trend was preserved at *t* = 30 min and *t* = 60 min for ciprofloxacin, in response to gentamicin the proportion of IDP-DEGs and the proportion of ordered DEGs was approximately equal (Table 3). Seventeen GEN-specific and 30 CFX-specific IDP-DEGs were discerned from these profiles, with an overlap of 51 shared IDPs (Table S2). This suggests that antibiotic-induced IDP differential expression is mechanism-specific.

**Table 3:** Number of IDP-DEGs vs Ordered Protein-DEGs between Resistant and Susceptible *E. coli* Isolates at Each Timepoint in Response to 1 mg/L Ciprofloxacin and 4 mg/L Gentamicin^a^

time point	ciprofloxacin (CFX)		gentamicin (GEN)	
	IDP-DEGs	ordered DEGs	IDP-DEGs	ordered DEGs
*t* = 0 min	2 (1.3% of IDPs)	2 (0.2% of ORD)	1 (0.7% of IDPs)	3 (0.2% of ORD)
*t* = 10 min	46 (30.1% of IDPs)	178 (14.0% of ORD)	13 (8.6% of IDPs)	52 (4.1% of ORD)
*t* = 30 min	55 (35.9% of IDPs)	228 (17.9% of ORD)	55 (36.4% of IDPs)	413 (32.4% of ORD)
*t* = 60 min	47 (30.7% of IDPs)	203 (15.9% of ORD)	54 (35.8% of IDPs)	418 (32.8% of ORD)

a Recorded DEGs are a combination of both up- and downregulated genes.

In order to further illustrate the potential role of intrinsic disorder in the evolution of AMR, we discuss below two proteins in which IDR mutations directly influenced the emergence of either resistance or susceptibility in bacteria, namely: the 50S ribosomal protein L2 (rplB) in *Bacillus subtilis* and the oxidoreductase aegA in *E. coli*.

**Figure 5:**
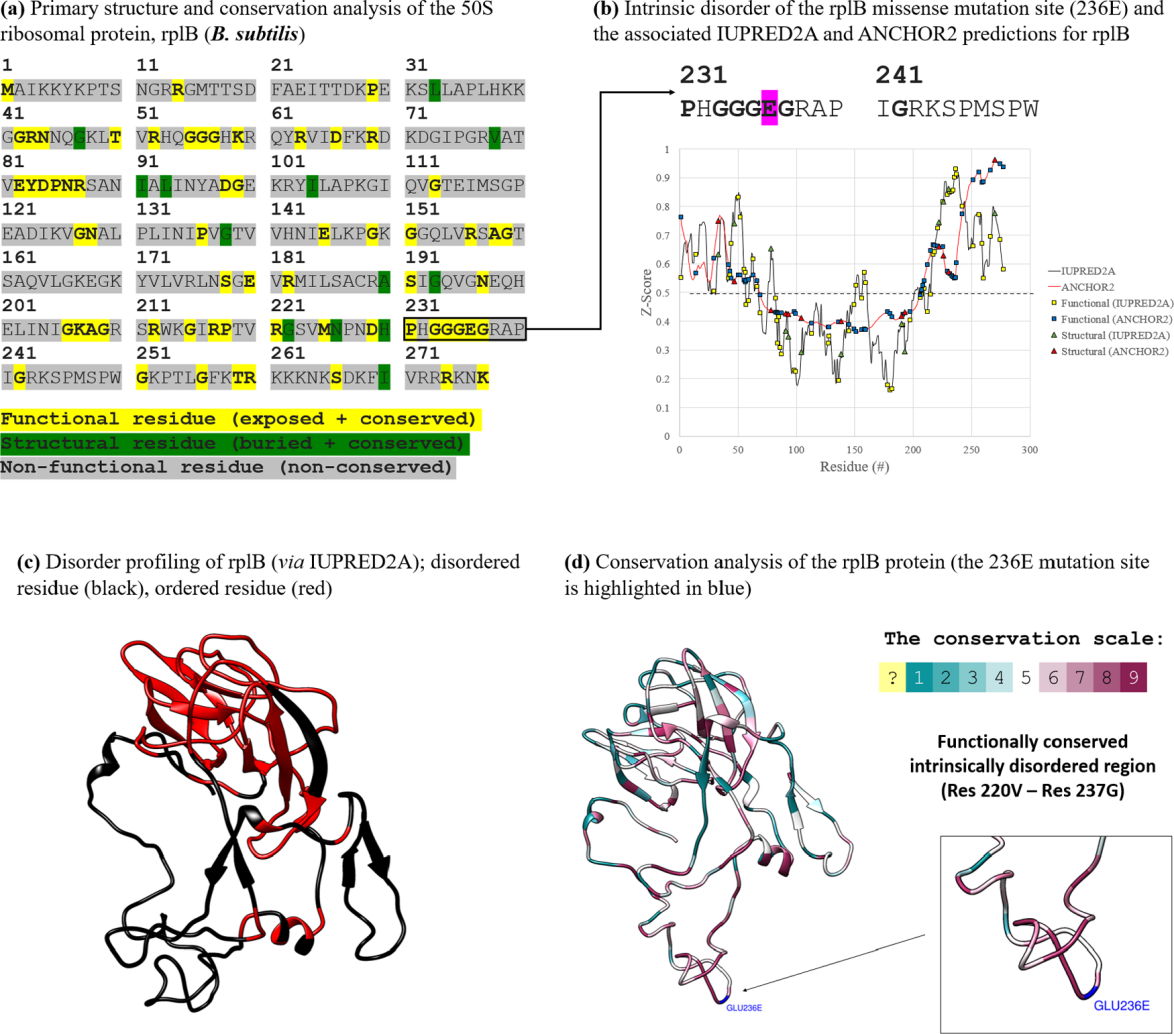
(a) Conservation analysis (obtained from CONSURF^20^) of the rplB protein in *Bacillus subtilis* mapped to its primary structure–individual residues is classified as functional if they are predicted to be conserved and solvent exposed, (b) an exploratory analysis of the amino acid sequence of rplB using IUPRED2A^9^ and ANCHOR2 reveals the extensive disorder of its C- and N-terminus, (c) IUPRED2A disorder profiling mapped to a representative structure of rplB (derived from PDB accession**:** 7aqc, in complex with 23S rRNA (not shown)), and (d) conservation analysis of rplB in *Bacillus subtilis* (strain 168) mapped to its 3D structure (mutation site–E236–highlighted in blue).

*Burkholderia thailandensis*, a Gram-negative rod, produces a unique polyketide-peptide called bactobolin. Polyketides, secondary metabolites produced by a range of organisms (including bacteria and fungi), exhibit excellent antimicrobial activity.^29^ Mirroring the action of other antimicrobials, bactobolin binds to the bacterial ribosome. Bactobolin resistance does not impede the action of alternative ribosomal therapeutics owing to its unique, conserved binding partner, the 50S ribosomal protein, L2 (encoded by the gene *rplB*).^30^ The whole-proteome profiling calculations outlined in Table 2 reveal that rplB is an intrinsically disordered protein. A previous study detected that bactobolin-resistant strains of *Bacillus subtilis* each presented with a mutation in the *rplB* gene, modifying the structure of the expressed L2 protein. This conferred resistance against bactobolins A and C, two potent antimicrobial polyketides isolated from *B. thailandensis*. Although it was not identified by the original authors, both resistance-mediating missense mutations (E236A and E236Q) occur within a functionally conserved, intrinsically disordered region of the protein implicated in DnaA binding.^31^ These C-terminus modifications were found to substantively influence the minimum inhibitory concentration of the *B. subtilis* mutant cell line, demonstrating that structural modifications induced by IDR mutations can mediate the emergence of AMR in the case of bactobolin polyketides.^32^ The conservation analysis and disorder profiling of rplB is given in Figure 5.

**Figure 6:**
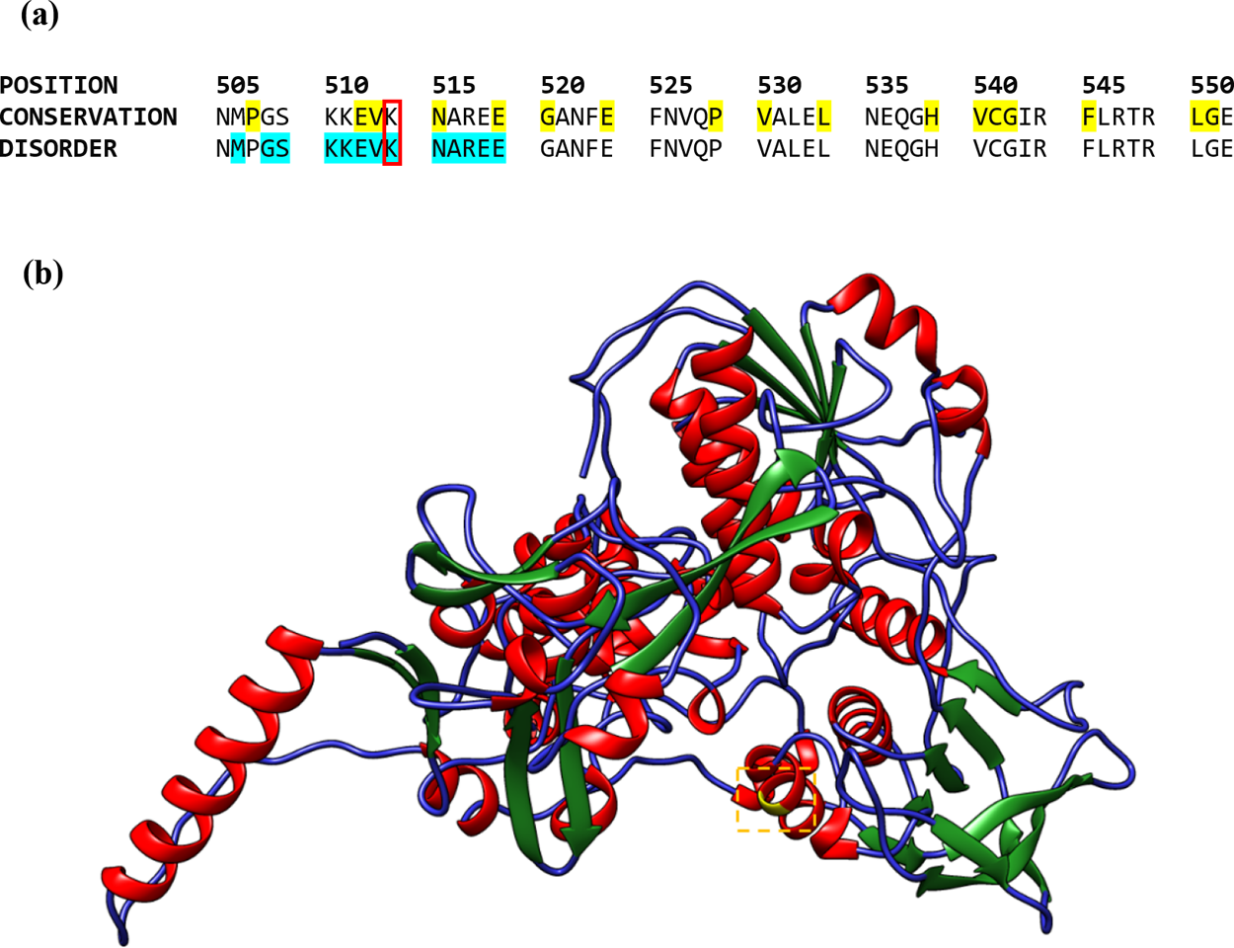
(a) Partial conservation and disorder profile of the *E. coli* oxidoreductase, *aegA*. Conserved residues (obtained from COBALT^18^) are highlighted in yellow, disordered residues (obtained from IUPRED2A^9^) are highlighted in cyan, and the K514 residue is highlighted in red. (b) 3D structure of *aegA* (K514R mutation site highlighted in yellow, AlphaFold-predicted structure^34^).

Mutations within functionally conserved IDRs reflect a major proponent of the relationship between intrinsic disorder and AMR. However, shorter disordered patches can also influence the response of the bacteria to an antibiotic. For example, *aegA* encodes an *E. coli* oxidoreductase involved in the breakdown of uric acid. It presents several long IDRs (with a maximum length of 46 AA residues). Surprisingly, a K514R amino acid substitution in a short disordered patch (12 residues in length) induced by an A1541G missense mutation was reported to double the sensitivity of a commensal *E. coli* isolate to enrofloxacin.^33^ A conservation analysis of *aegA* indicates that the K514 residue is neither functional nor highly conserved (Figure 6). The increased sensitivity arising from this IDR mutation supports that (1) IDR mutations can influence both antimicrobial susceptibility and resistance and (2) the length and sequence conservation of an IDR are not the sole determinants of its biological significance.

### Identifying Novel Drug Targets

The disorder profiles calculated in Table 2 can be overlaid with multiomics data sets to provide new insights into the expression, regulation, and functional importance of bacterial IDPs. As a demonstrative example, the disorder profile of the *E. coli* K12 MG1655 proteome was combined with experimental data assessing the antimicrobial susceptibility of putative single-gene knockout (KO) cell lines.^35^ A total of 23 IDPs were identified whose knockout-induced susceptibility to one or more antibiotics, lending support to a relationship between bacterial intrinsic disorder and AMR (Table 4). Transposon-directed insertion site (TraDIS) sequencing data from Goodall et al.^36^ was also reanalyzed through the lens of intrinsic disorder, revealing the disordered “survivasome” of *E. coli* K12 BW25113—the set of all IDPs essential for the growth and viability of the cells. Essential proteins are a common antimicrobial drug target,^37^ and thus, the IDPs identified in Table 5 could potentially be used to combat antibiotic-resistant variants of this *E. coli* strain.

**Table 4:**
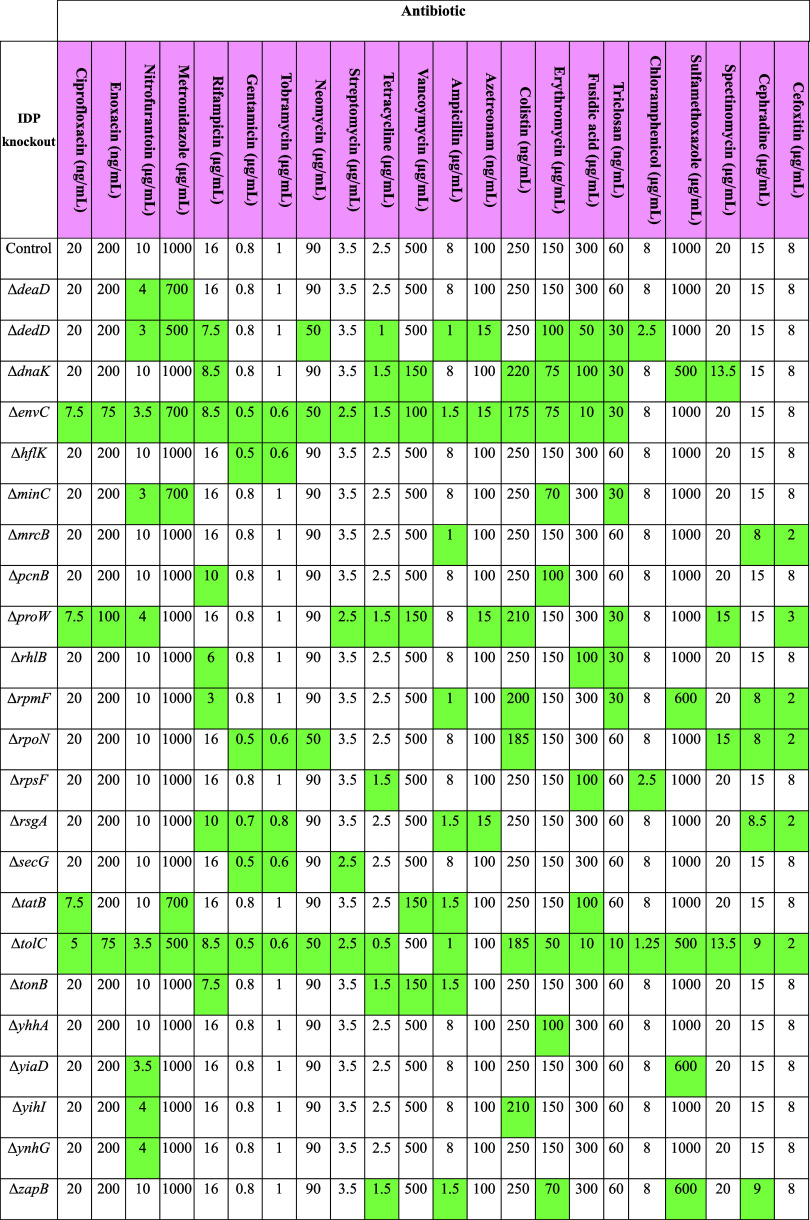
Minimum Inhibitory Concentrations of *E. coli* K12 MG1655 IDP Knockouts (KO)^a^

a Knockouts with a reduced MIC against a particular antibiotic when compared to the wild-type are shaded in green (adapted from Liu et al.^35^).

**Table 5:** Disordered Survivasome of *E. coli* K12 BW25113 (*N* = 17)^a^^,^^b^

IDP	log likelihood score	number of residues	number of disordered residues	percentage intrinsic disorder (%)	maximum IDR length
*dnaA*	–22.84	467	56	11.99	44
*dnaX*	–20.27	643	154	23.95	117
*ftsH*	–4.15	644	88	13.66	55
*ftsY*	–26.72	497	179	36.02	178
*grpE*	–5.77	197	63	31.98	52
*gyrA*	–19.59	875	47	5.37	32
*infB*	–14.55	890	329	36.97	280
*mreC*	–6.71	367	92	25.07	92
*prfA*	–19.80	360	142	39.44	34
*rplB*	–20.69	273	157	57.51	61
*rplO*	–172.16	144	48	33.33	31
*rpmH*	–172.16	46	46	100.00	46
*rpoD*	–28.13	613	149	24.31	49
*sucB*	–6.84	405	120	29.63	51
*topA*	–13.66	865	155	17.92	32
*yffS*	–7.28	269	74	27.51	36
*zipA*	–14.84	328	182	55.49	160

a Calculated descriptors of intrinsic disorder derive from the IUPRED2A-based whole-proteome profiling analysis, as shown in Table 2.

b Genes with a log likelihood score less than log_2_(12) belong to the essential gene mode, with more negative values indicating a greater confidence that the gene is essential (adapted from Goodall et al.^36^).

## Conclusions

The detailed bioinformatics analysis presented here uncovered a previously unknown relationship between intrinsic bacterial disorder and antimicrobial resistance. Genes encoding disordered proteins were found to be differentially expressed in response to antibiotic-induced cellular stress, highlighting the importance of intrinsic disorder for the functioning of the bacterial stress response. Genes encoding proteins with long IDRs are differentially expressed between resistant and susceptible bacterial strains following antibiotic exposure. Despite their lower incidence rate (compared to eukaryotic IDPs), bacterial IDPs were shown to be conserved at an amino acid level, to induce antimicrobial susceptibility on knockout, and to present with IDR mutations that directly contribute to both antimicrobial susceptibility and AMR. These findings lay the foundation for a relationship between IDPs and antibiotic resistance and support a new approach to antimicrobial drug discovery.

## Supporting Information Available

Whole-proteome profiling parameters, DGE data for ciprofloxacin (1, 2 mg/L) and gentamicin (4 mg/L), and functional interaction networks for IDP-DEGs (PDF)
