# Supplementary material for: Targeting Protein Disorder for the Remediation of Antimicrobial Resistance
Source: ACS Omega. 2024 Dec 10;9(51):50589–98. doi: 10.1021/acsomega.4c08427 (PMC11683595; doi:10.1021/acsomega.4c08427)
Supplement: Supplementary file 1 — ao4c08427_si_001.pdf [file ao4c08427_si_001.pdf]

Targeting protein disorder for the remediation of antimicrobial resistance

Jack O' Callaghan[1,2,3], Michael P Ryan[4], Sarah Hudson[2,3,5*] and Damien Thompson[1,2,3,5*]

[1]Department of Physics, Bernal Institute, University of Limerick, Limerick, V94 T9PX, Ireland

[2]Bernal Institute, University of Limerick, V94 T9PX, Ireland.

[3]Science Foundation Ireland Research Centre for Pharmaceuticals (SSPC), University of Limerick, V94 T9PX, Ireland.

[4]Department of Applied Sciences, TUS Midwest, Limerick, V94 EC5T, Ireland

[5]Department of Chemical Sciences, Bernal Institute, University of Limerick, Limerick, V94 T9PX, Ireland

*Correspondence should be addressed to: damien.thompson@ul.ie (Prof. Damien Thompson); sarah.hudson@ul.ie (Prof. Sarah Hudson)

Table S1: Parameters calculated during whole-proteome profiling analysis using IUPRED2A

Parameter	Description
Gene name	An identifier unique to the protein being analysed. For example, “GN=rpmF” appears in the first line of the FASTA file for the bacterial ribosomal protein, rpmF.
Number of residues	The primary structure of the protein is encoded as a string of characters in the FASTA file. The number of characters in the string equates to the number of amino acid residues in the protein.
Number of disordered residues	IUPRED2A gives a residue-wise prediction of disorder, which is expressed as a Z-score. This score ranges from 0 to 1. Residues were classified as disordered if they had a Z score greater than 0.5.
Percentage intrinsic disorder (%)	The percentage intrinsic disorder is the quotient of the number of disordered residues and the total number of residues.
Number of disordered binding residues	IUPRED2A performs a secondary analysis called ANCHOR2, which predicts disordered residues that are involved in binding. Disordered residues with an ANCHOR2 Z-score greater than 0.5 were classified as disordered binding residues.
Percentage binding IDR (%)	This is the quotient of the number of disordered binding residues, and the total number of residues.

Number of disordered regions	A continuous string of disordered residues defines an intrinsically disordered region (IDR) in a protein. An IDR is bounded by residues with an IUPRED2A Z-score < 0.5.
Maximal IDR length	The number of disordered regions is predicted, and the length of each region is calculated. The IDR of the greatest magnitude is returned as the maximal IDR length.
Maximal IDR contribution to protein disorder (%)	This is the quotient of the maximal IDR length and the total number of disordered residues. This frames the importance of the IDR in the context of the protein's composition.

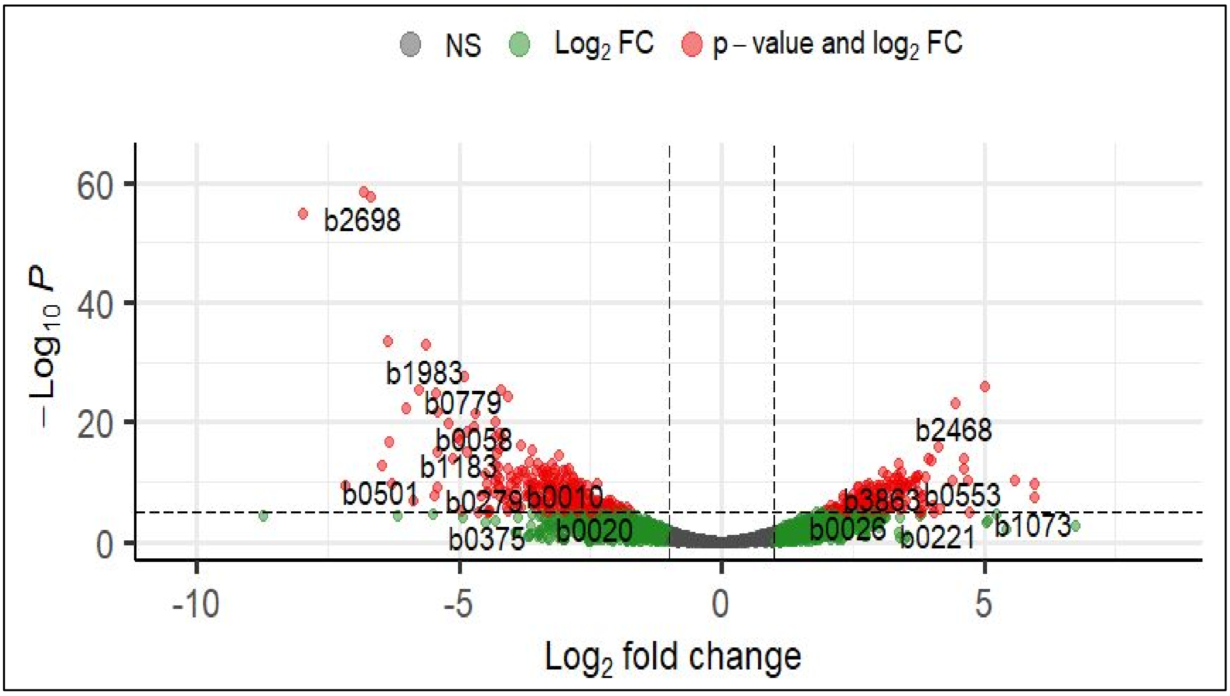

Figure S1: Volcano plot of *E. coli* K12 MG1655 genes differentially expressed in response to 2 µg/mL of ciprofloxacin. Genes with a negative log-fold change are upregulated in antibiotic-exposed cells, and genes with a positive log-fold change are downregulated[1].

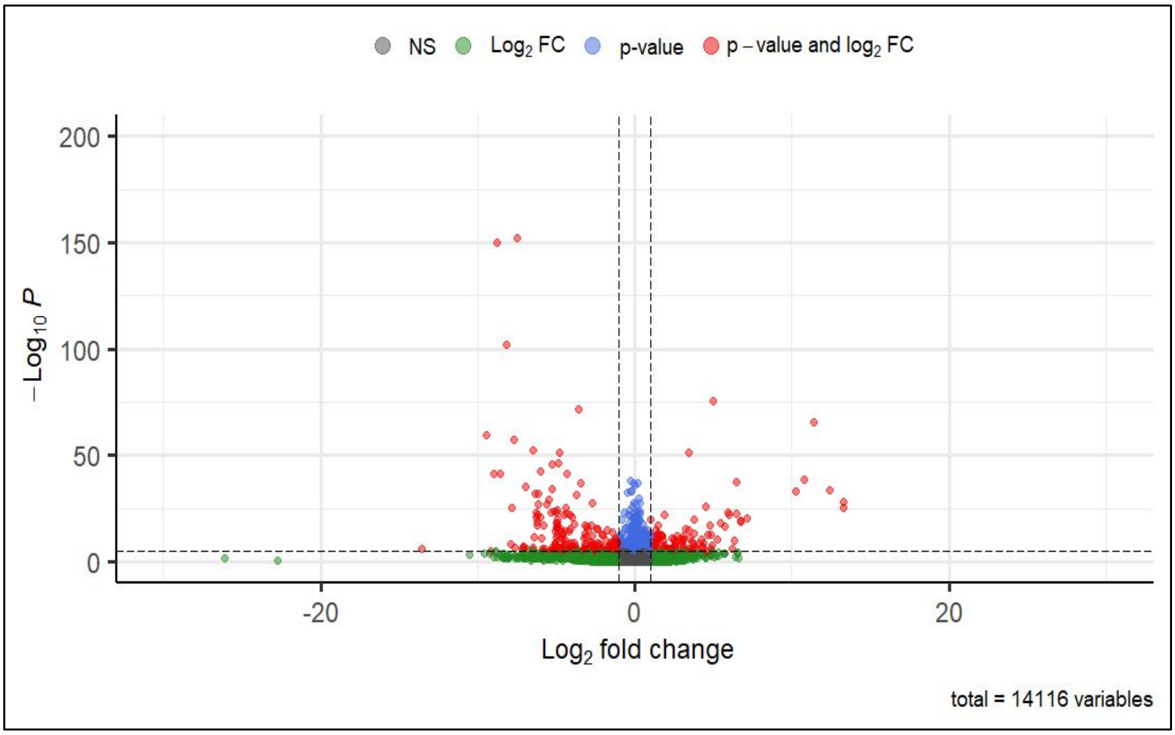

Figure S2: Volcano plot of differential gene expression data across all timepoints for susceptible and resistant *E. coli* isolates in response to 1 mg/L of ciprofloxacin. Genes with a negative log fold change represent genes that are downregulated in susceptible strains, while genes with a positive log fold change are upregulated[2].

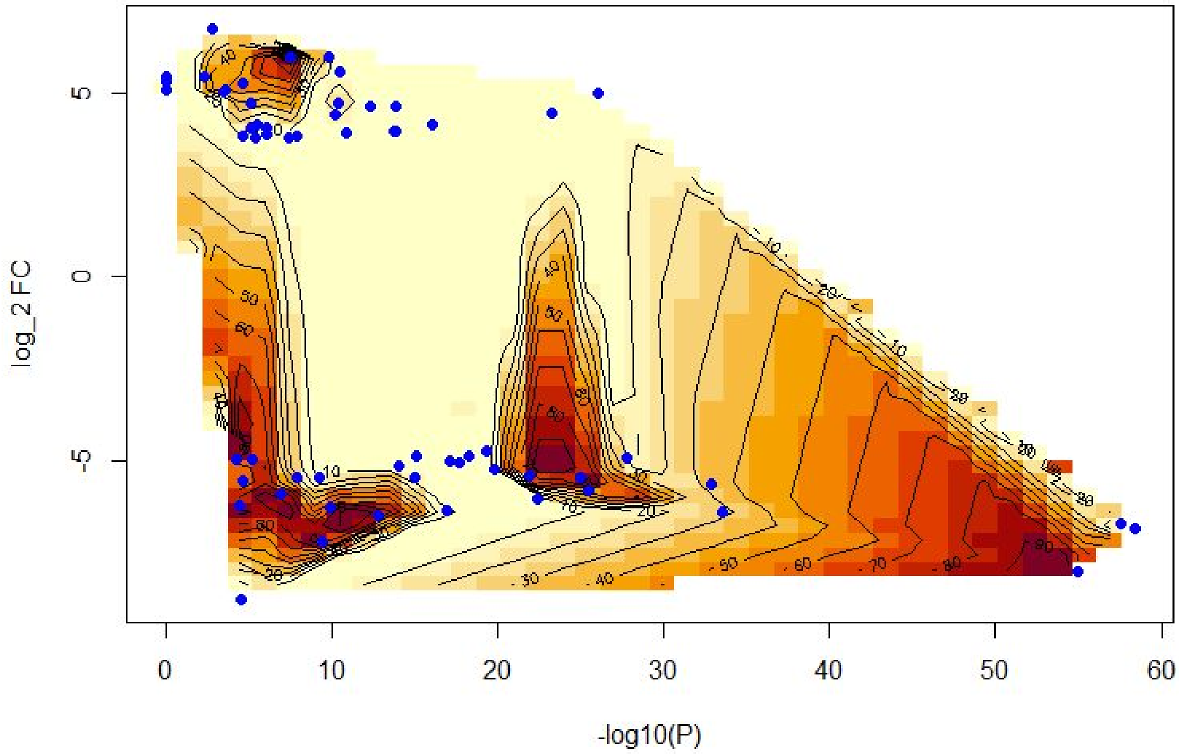

Figure S3: Variation in the log fold change (**log_2FC**) and adjusted p-value (**-log10(P)**) of the Top 60 DEGs for wild-type *E. coli* K12 MG1655 in response to 2 μg/mL of ciprofloxacin[1]. The blue dots correspond to DEGs. The overlayed contour plot reflects the percentage intrinsic disorder of the DEG-associated protein, as predicted by ESPRITZ. Darker regions correspond to proteins with a greater percentage disorder. Genes with a positive log-fold change are downregulated following antibiotic exposure.

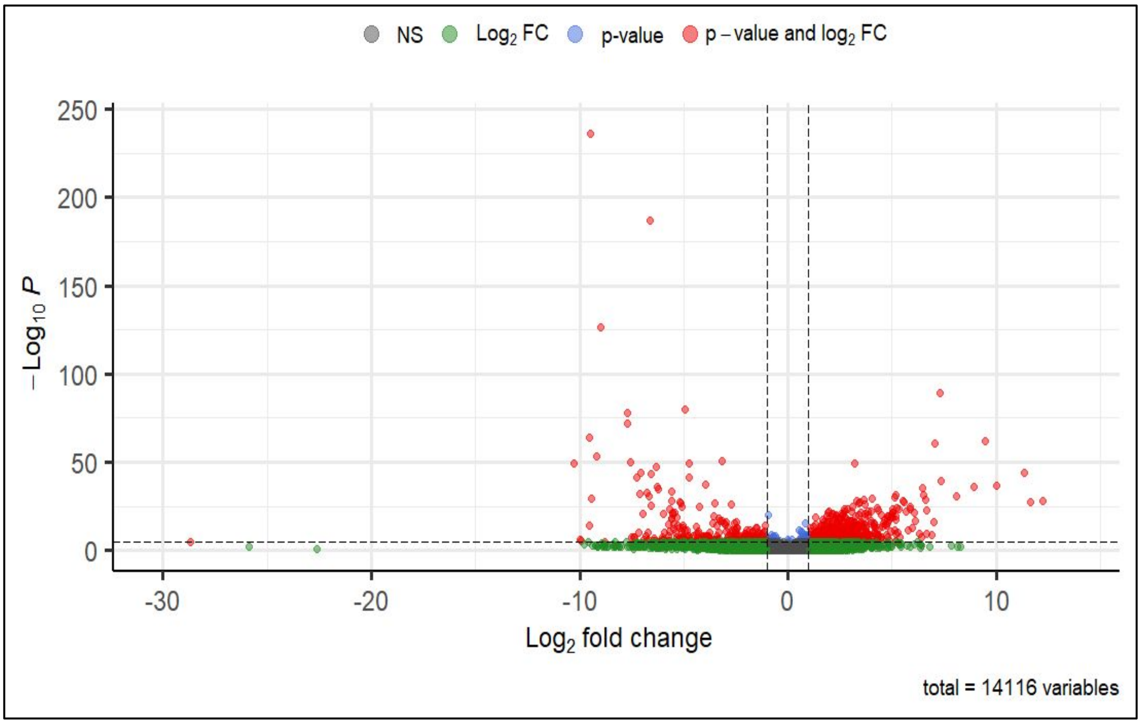

Figure S4: Volcano plot of differential gene expression data across all timepoints for susceptible and resistant *E. coli* isolates in response to 4 mg/L of gentamicin. Genes with a negative log fold change represent genes that are downregulated in susceptible strains, while genes with a positive log fold change are upregulated[2].

(a)

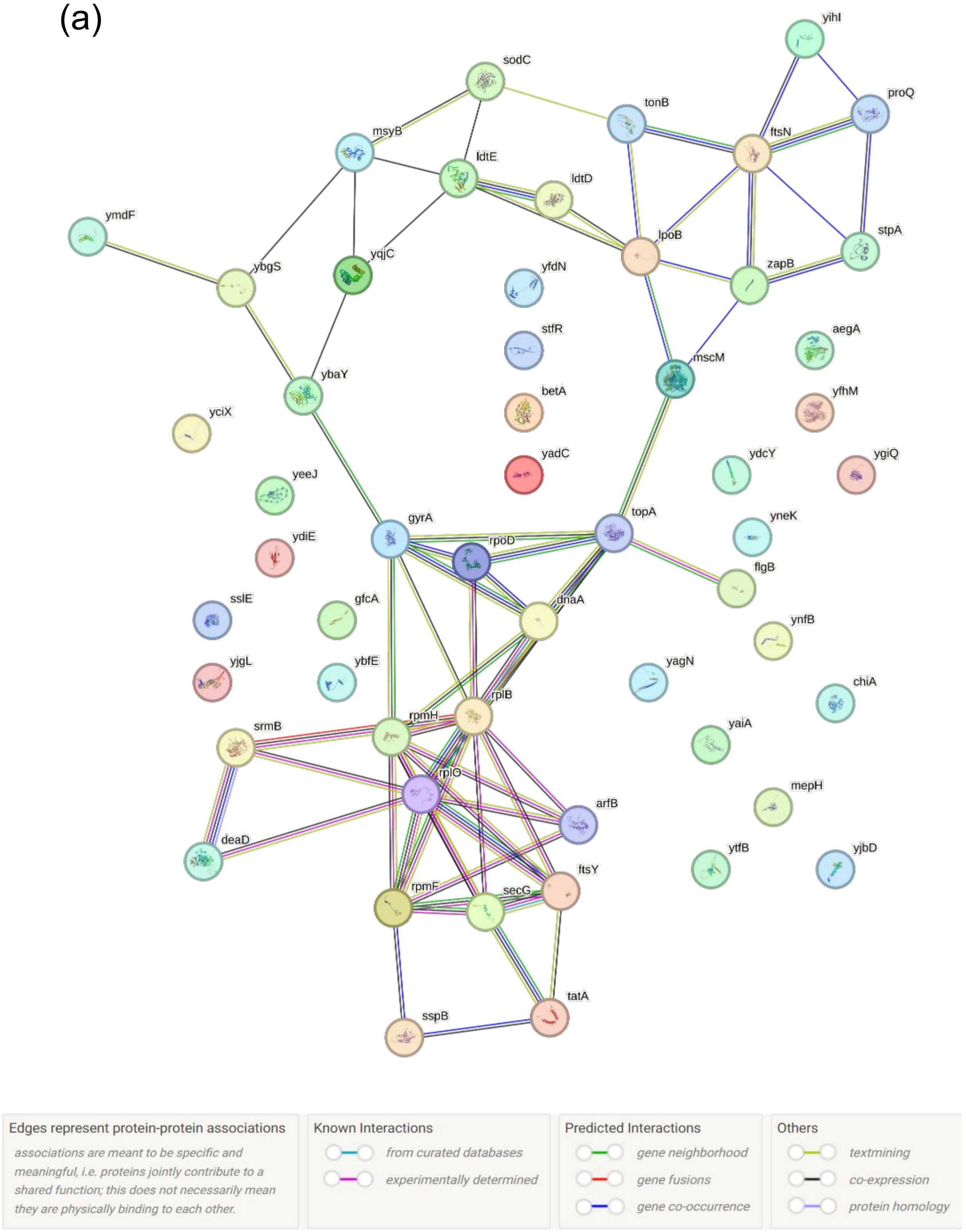

Figure S5 (a): Functional interaction network for IDPs differentially expressed between resistant and susceptible isolates in response to 1 mg/L of ciprofloxacin. Edges within each network represent functional and physical interactions between each of the proteins[2].

(b)

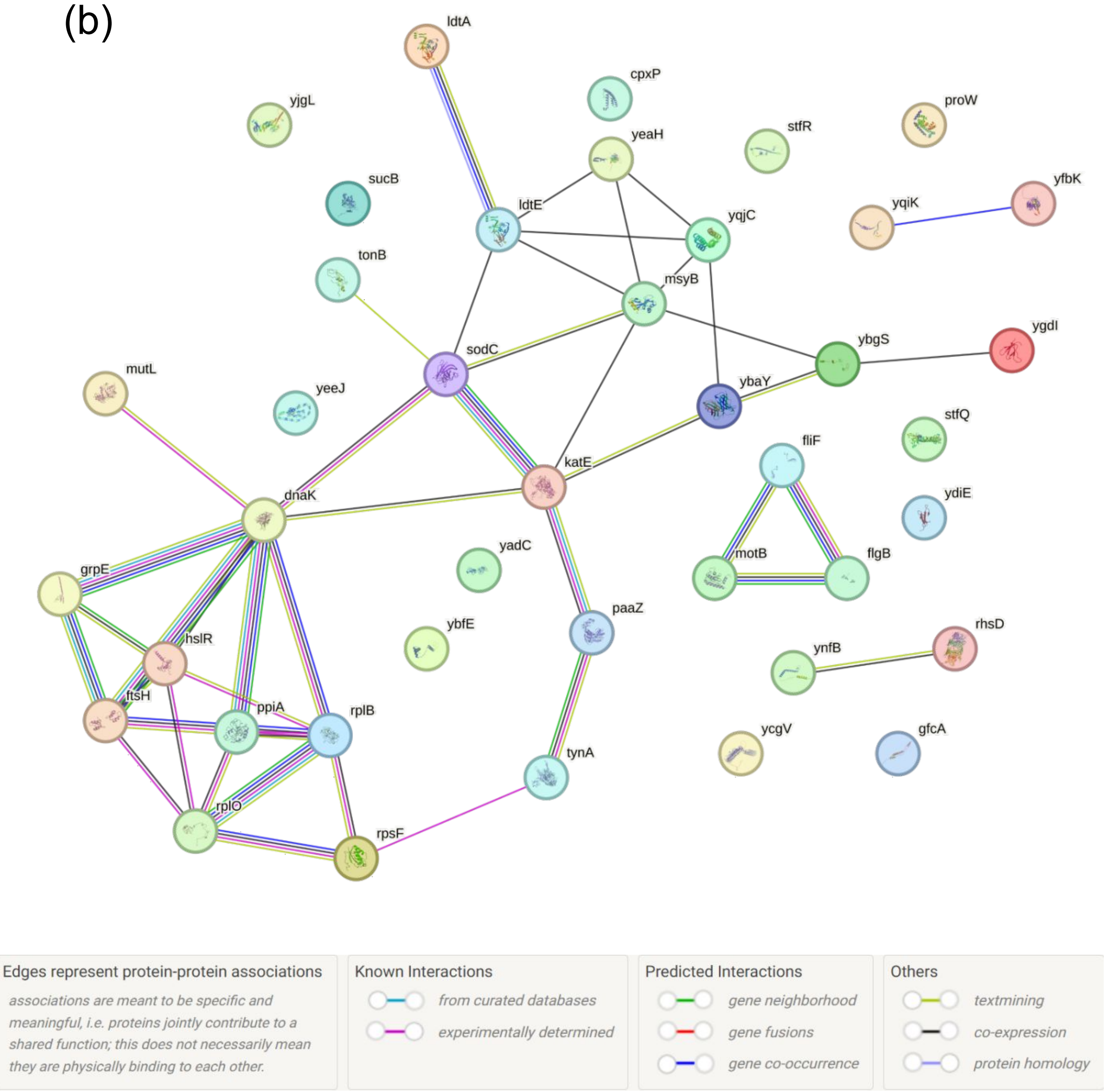

Figure S5 (b): Functional interaction network for IDPs differentially expressed between resistant and susceptible isolates in response to 4 mg/L of gentamicin. Edges within each network represent functional and physical interactions between each of the proteins[2].

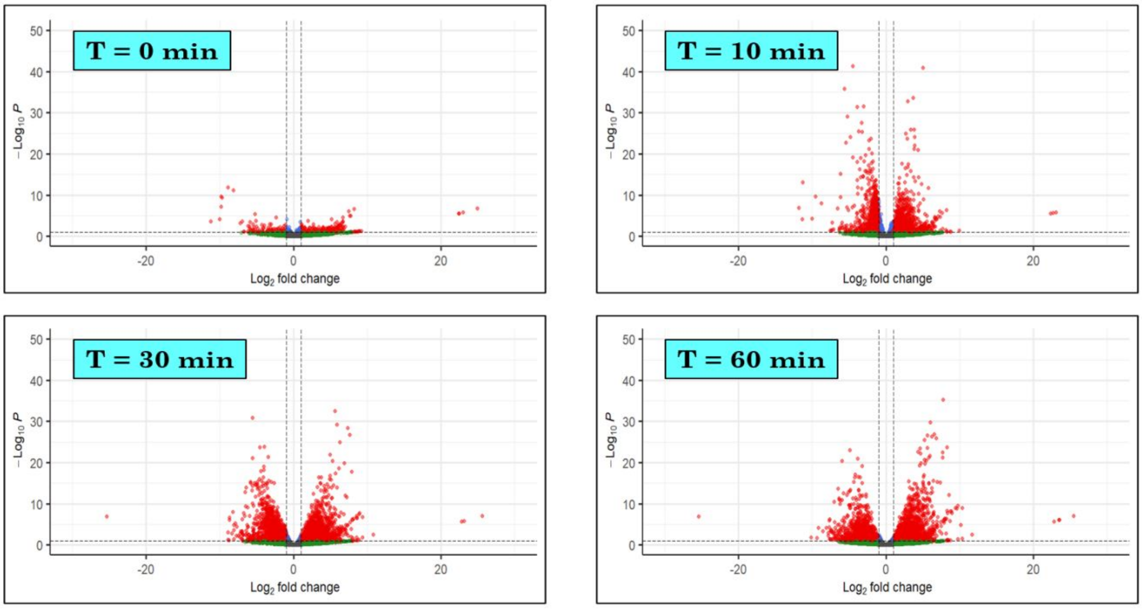

Figure S6: Differentially expressed genes for *E. coli* / CFX at each timepoint (R vs. S, treated)[2]

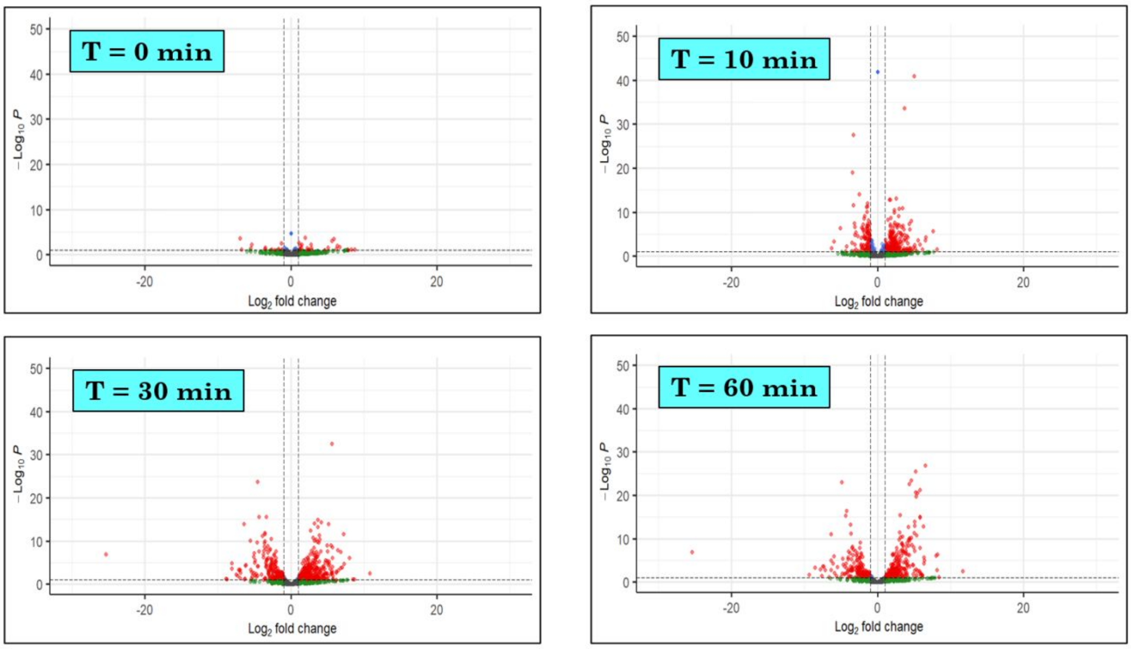

Figure S7: Differentially expressed ordered proteins for *E. coli* / CFX at each timepoint (R vs. S, treated)[2]

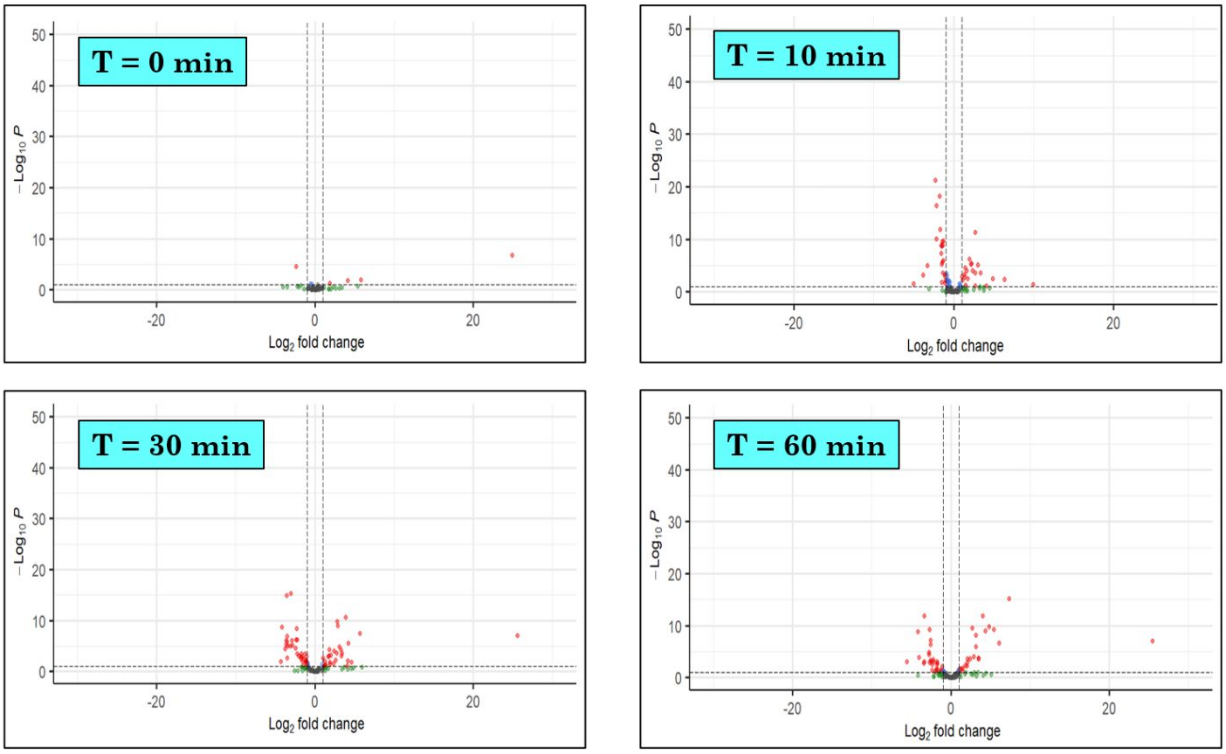

Figure S8: Differentially expressed IDPs for *E. coli* / CFX at each timepoint (R vs. S, treated)[2]

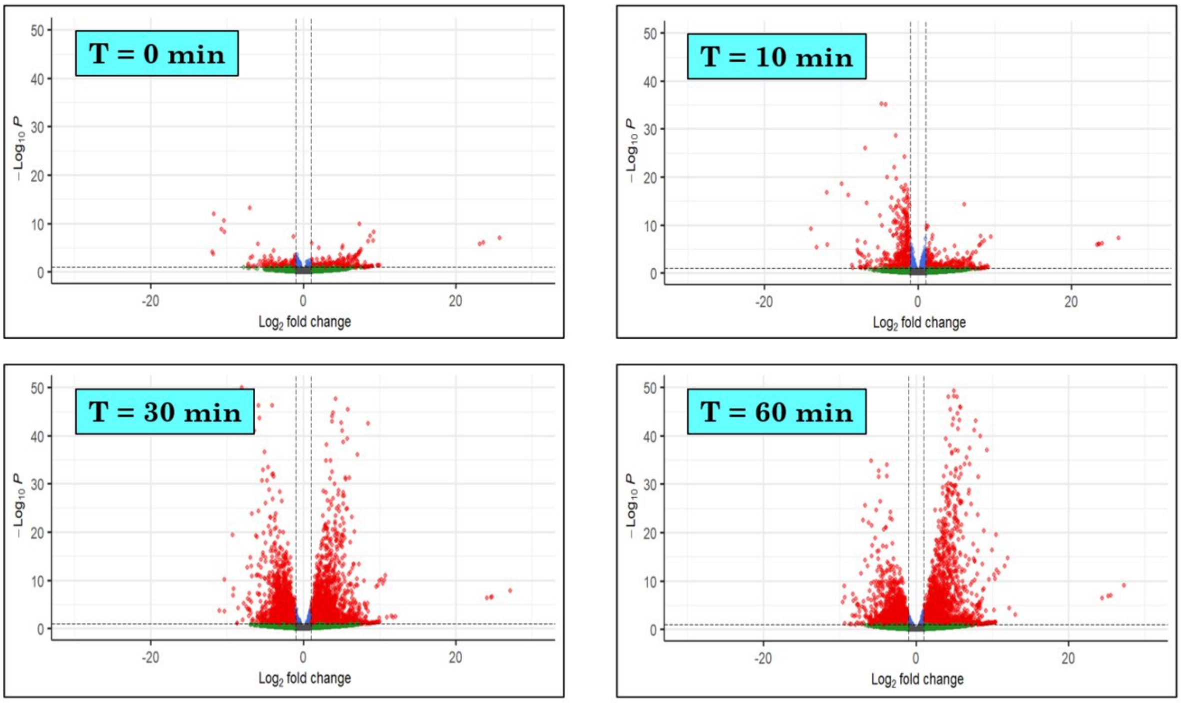

Figure S9: Differentially expressed genes for *E. coli* / GEN at each timepoint (R vs. S, treated)[2]

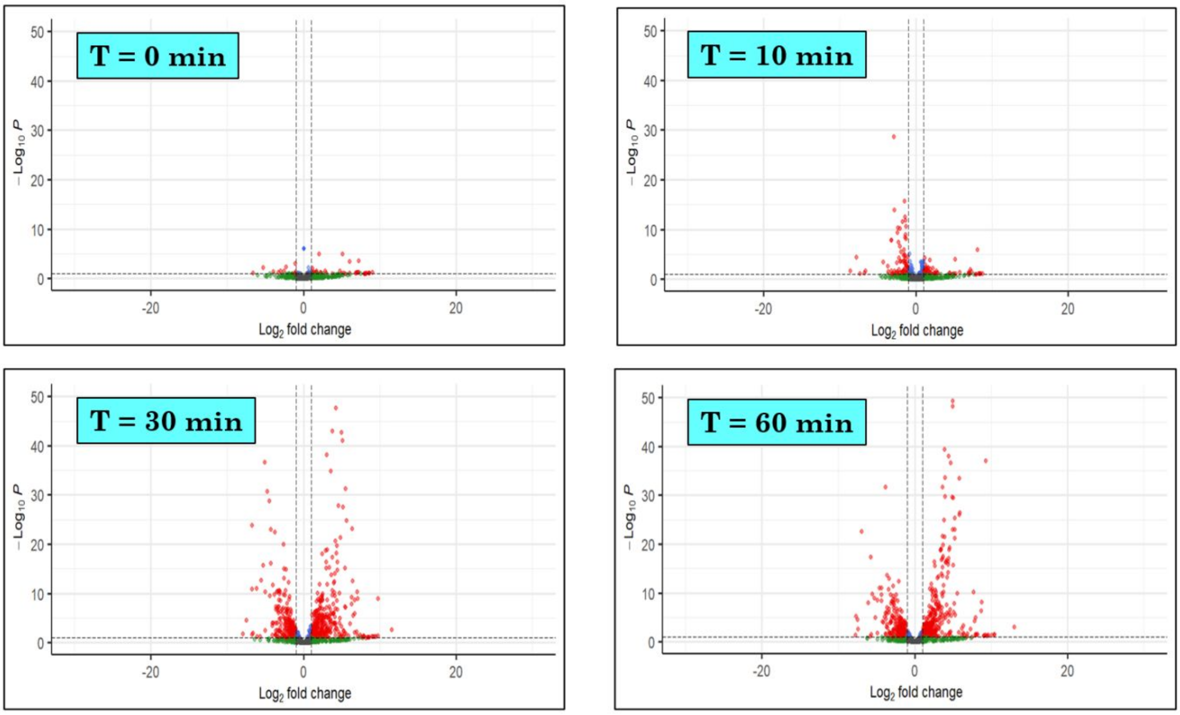

Figure S10: Differentially expressed ordered proteins for *E. coli* / GEN at each timepoint (R vs. S, treated)[2]

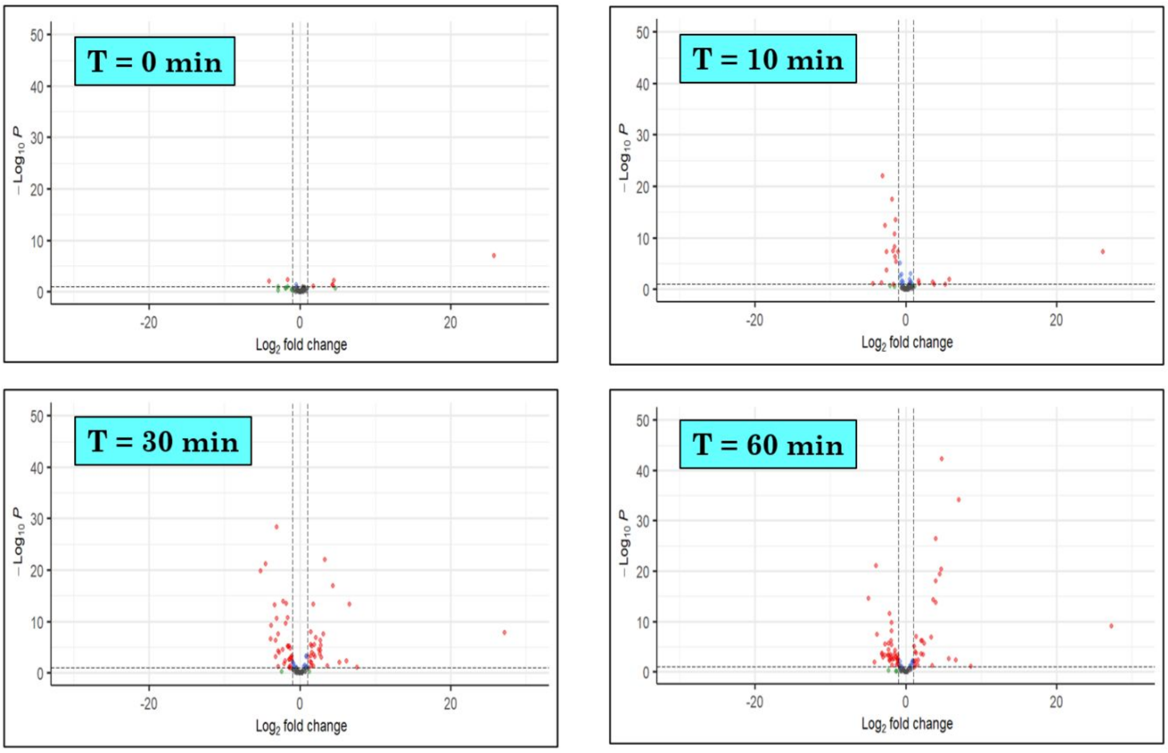

Figure S11: Differentially expressed IDPs for *E. coli* / GEN at each timepoint (R vs. S, treated)[2]

Table S2: Time-dependent differential expression of *E. coli* IDPs for resistant and susceptible isolates in response to 1 mg/L of ciprofloxacin and 4 mg/L of gentamicin. Genes differentially expressed at a given timepoint are shaded black[2].

	**Ciprofloxacin**				**Gentamicin**			
**IDP**	**T = 0 min**	**T = 10 min**	**T = 30 min**	**T = 60 min**	**T = 0 min**	**T = 10 min**	**T = 30 min**	**T = 60 min**
aegA		■	■				■	
amiB		■	■					
betA	■	■		■		■		■
clpA							■	
cyoA				■				
deaD			■	■			■	■
dedD		■						■
dnaA		■	■	■			■	■
dnaK		■		■		■	■	■
dnaX			■	■				
envC			■					
erfK		■	■	■			■	■
ftsH			■			■	■	■
ftsK		■	■				■	
ftsN								■
ftsY			■	■			■	■
gfcA	■			■	■	■	■	■
grpE		■				■	■	■
gyrA		■	■	■				
hflK				■				
hslR						■	■	■
hypB		■					■	■
infB							■	■
katE				■			■	■
lpoA		■	■					
lpoB		■	■	■			■	■
mepA							■	
mepH		■	■	■				

minC		■	■	■				
mrcA			■	■			■	■
mrcB								■
mreC			■					
mscM			■				■	
msyB			■	■			■	■
mutL			■	■			■	■
nagZ		■						
nlpD		■	■					
pcnB			■	■			■	■
ppiA		■						
prfA		■	■				■	■
proQ							■	■
proW								■
ptrA		■	■					
rhlB		■						
rhlE			■	■			■	■
rhsD							■	■
rlpA		■					■	■
rodZ							■	
rluF		■						
rmuC		■	■	■				
rplB		■	■	■		■	■	■
rplO		■	■	■		■	■	■
rpmF		■	■				■	
rpmH		■	■	■			■	■
rpoD		■	■			■	■	
rpoN							■	■
rpsF		■	■	■		■	■	■
rsxC			■	■				
sbcC			■				■	■
secG		■	■	■				
slyD		■	■	■				
sodC			■	■			■	■
srmB		■	■	■			■	

sspB		■	■	■			■	■
stfR		■					■	
stpA							■	■
sucB		■					■	■
tatA			■					
tatB			■					
tolA			■				■	■
tolC								■
tonB							■	■
topA		■				■		
yaaX				■				■
yaiA				■				■
ybaY			■					
ybfE		■	■	■			■	■
ybgS			■	■			■	■
yciX		■	■	■				
ydbA		■						
ydiE							■	■
yeaH				■			■	■
yfdN		■		■				
yfhM		■	■	■			■	■
yfiF		■	■					
ygdI			■	■			■	■
yheS		■	■	■				■
yhfK		■						
yhhA			■	■			■	■
yiaD			■	■				
yihI		■	■	■				■
yjbD				■			■	■
yjgL						■	■	■
ymdF				■				
ynfB						■	■	■
ynhG			■	■			■	■
yqiK								■
yqjC			■					

References

1. Sun, R., et al., *Genome-Wide Screening and Characterization of Genes Involved in Response to High Dose of Ciprofloxacin in Escherichia coli.* Microbial Drug Resistance, 2022: p. 501-510.
2. Bhattacharyya, R.P., et al., *Simultaneous detection of genotype and phenotype enables rapid and accurate antibiotic susceptibility determination.* Nat Med, 2019. **25**(12): p. 1858-1864.
